# Is the retromandibular approach a suitable approach to anatomical reduction of unilateral subcondylar fracture? A non-randomized clinical trial

**DOI:** 10.1186/s13005-022-00342-1

**Published:** 2022-12-02

**Authors:** Abdo Ahmed Saleh Mohamed, Guangxin Rao, Lianxi Mai, Karim Ahmed Sakran, Saddam Noman Al-wesabi, Chaobin Pan, Zhaoyu Lin

**Affiliations:** 1 Department of Oral & Maxillofacial Surgery, Sun Yat-Sen Memorial Hospital, Sun Yat-Sen University, Guangzhou, China; 2 Department of Oral and Maxillofacial Surgery, Faculty of Dentistry, Ibb University, Ibb, Yemen; 3 Department Oral and Maxillofacial Surgery, Lanzhou University First Affiliated Hospital, Lanzhou University, Lanzhou, Gansu Province, China; 4 Resident, State Key Laboratory of Oral Diseases and National Clinical Research Center for Oral Diseases and Department of Oral and Maxillofacial Surgery, West China Hospital of Stomatology, Sichuan University, Chengdu, China; 5 Department of Oral and Maxillofacial surgery, School of stomatology, Lanzhou University, Lanzhou, Gansu, China

**Keywords:** Condylar fracture, Retromandibular approach, Reduction accuracy, Three-dimensional CBCT analysis

## Abstract

The aim of this study was to evaluate the efficacy of the retromandibular approach (RMA) to produce three-dimensional (3-D) reduction of the unilateral subcondylar fracture and Temporomandibular Joint (TMJ) functional implication.

**Methods:** A prospective cohort study was designed. Twenty-nine patients with unilateral subcondylar fracture underwent consecutively Open Reduction, and Internal Fixation. The cohorts were divided into two groups; RMA group (*n* = 16, 55.17%) and submandibular approach SMA group (*n* = 13, 44.82%). The primary outcome was the anatomical 3-D reduction of the condyle. The secondary outcome was to compare the condyle position and inclination finding with TMJ outcomes. Helkimo Index score was used to evaluate the TMJ outcome at six months postoperatively.

**Result:** There was a significant difference between the mediolateral condylar inclination, condylar medial and vertical positions when RMA compared with SMA groups (*P* < 0.05). The medial joint space was correlated with the medial condylar position in both groups (*P* < 0.05). The Helkimo Ai and Di was associated with mediolateral condylar inclination in SMG; however, Helkimo Ai was found to be correlated with the RMA group.

**Conclusion:** The current study demonstrates that the RMA could re-establish the anatomical position of the unilateral subcondylar fracture in patients undergoing ORIF. The clinical outcome of the TMJ with RMA was better than SMA.

**Supplementary Information:**

The online version contains supplementary material available at 10.1186/s13005-022-00342-1.

## Background

Condyle fracture is one of the most fractures in the mandibular bone. It ranges from 25 to 50% of the mandibular fracture [1, 2]. The location and the direction of the fracture line might determine the classification of the fracture. In general, condyle fracture has been classified based on the anatomical location of the fracture line into three types; condyle head, condyle neck, and condyle base (subcondylar) fractures [3]. The treatment for condyle fracture varies from the closed condyle treatment (CCT) to the open reduction and internal fixation (ORIF). Each approach has its own set of pros and cons; however, this is still a topic of controversy [2, 4]. The most hazardous effect with the open approach is the facial nerve injury, whereas in the closed approach, is the ability to reduce the condyle to its normal position. A fracture line that starts above the sigmoid notch and, 2/3 of it, passes down the sigmoid notch is called subcondylar fracture.

The treatment of the subcondylar fracture has been debated for a long time; however, recently, there has been a trend to be treated by open reduction and internal fixation [5, 6]. ORIF is performed to get direct access to the fracture line. Therefore, many surgical approaches have been developed to manage the subcondylar fracture, for instance, submandibular, high submandibular, retromandibular, and intraoral (with or without endoscopic/transbuccal trocar) approaches. Although these approaches have the risk of jeopardizing the facial nerve function and other postsurgical complications, the high submandibular approach (HSMA) or retromandibular approach (RMA) were the safest approaches concerning minimizing the facial nerve injury [5].

Furthermore, the anatomical location and proximity of the subcondylar fracture to the TMJ can have a long-term functional impairment, especially if proper anatomical relationships are not accurately re-established.

Generally, anatomical reduction and stability of subcondylar fracture after ORIF are multifactorial issue, affected not only by the approach but also by the level of the fracture line, number and type of bone plates and also whether it is 2-D or 3-D plate. Plus, concomitant fractures and the way to fix it [7, 8]. Huang CM et, al. reported that placing the miniplates fixation in the posterior margin of the ascending ramus margin increases the fixation stability [9].

For that, providing an excellent surgical field is very important to have successful reduction. RMA and SMA approaches have been advocated to expose, operatively reduce, and fix these fractures. Previous studies concluded that both methods were able to provide reasonable access and comparable postoperative clinical results [10, 11]. Radiographically, few studies analyze the condyle position after ORIF. Our previous study has highlighted the condylar positional change with ORIF regardless of the surgical incision approach [12]. To the best of our knowledge, no paper discussed the condyle position, angulation, and joint space—anatomical reduction- between the RMA and the SMA for the subcondylar fracture. We hypothesized that the reduction of the condyle and the clinical outcome are not significantly different either by the RMA or SMA. This article aimed to answer the following questions:

1. Does the Retromandibular approach provide anatomical reduction for the unilateral condyle fracture over the submandibular approach?
2. Do the clinical outcomes differ with different approaches ?

This article used the CBCT to provide a detailed information related to the anatomical reduction of the subcondylar fracture and its clinical outcome in temporomandibular joint.

## Methods

### Study design

This prospective non-randomized cohort study was conducted at the 1st Hospital of Lanzhou University, Department of Oral and Maxillofacial Surgery, from September 2017 to February 2020. Twenty-nine consecutive patients who underwent ORIF (RMA or SMA) for the unilateral subcondylar fracture were included. All patients were evaluated presurgical with clinical examination and Panoramic/CT-scan. An informed consent form, including explanation about the two approaches, was obtained from patients. The cohorts were informed of the right to refuse to participate in the study or to withdraw consent to participate at any time without reprisal.

This study followed the Declaration of Helsinki on medical protocol and ethics and the regional Ethical Review Board of Stomatology College, Lanzhou University approved the study. The postoperative 3-D CBCTs were used for radiological evaluation. The clinical assessment was conducted by malocclusion, mouth opening limitation, and postoperative pain.

Comparing between retromandibular and submandibular approaches was implemented. Also, the fracture side and non-fracture side were analyzed.

The patients included in this study were suffering from simple unliteral subcondylar fracture, with 5° to 40**°** deviation between the subcondylar and the ascending ramus, more than 2 mm shortage of ramus, older than 18 years old, and limitation of mouth opening. Patients with any history of TMD, less than 18 years old, bilateral subcondylar fracture, condylar head fracture, insufficient dentition, treated with closed treatment or endoscopic and patients with comminuted fractures were excluded from this study. Demographic data is included in Table 1.

The Comprehensive AOCMF Classification System by Neff, A., et al., 2014 was used in this study [13].

### Clinical assessment

Helkimo index scoring system was implemented in the current study to evaluate TMJ function. Helkimo Ai has utilized the TMJ dysfunction subjectively. On the other hand, Di represented the objective assessment of the impaired TMJ function. Both subjective and objective symptoms were evaluated by the limitation of mouth opening, TMJ function impairment, pain in the muscle, and TMJ. The patient was classified as Ai0 (asymptomatic), AII (Mild symptoms), AiIII (severe symptoms). The score for the Di is shown in Table 2.

Patients were followed for six months (at least). Pain in the temporomandibular joint (TMJ) region, facial nerve weakness, occlusion disturbances, and interincisal mouth opening were assessed.

### Surgical operation

All the Open Reduction and Internal Fixation was performed by one surgeon in consecutive pattern. Patients underwent ORIF under general anesthesia. The RMA and SMA procedures are outlined below:

In the RMA group, it was similar to what Ellis and Dean described. Shortly, gentian violet was used to mark the subcutaneous skin incision (3–4 cm) below the ear pinna and 1 cm behind the angle of the mandible. Dissection was made until the subplatysmal layer. Once the parotid gland fascia was identified, the blind dissection was carried out parallel with facial nerve direction; once facial nerves were encountered, they were first carefully dissected and retracted to decrease the tension. The periosteum was incised at the posterior border of the mandibular. After the subperiosteal dissection of the ramus and subcondylar region, the reduction and fixation of the fracture fragments were obtained. For providing enough working space on the fracture line, the manual downward pressure was applied. Two suitable 2.0 mm miniplates were used to fix the fracture sides, and then copious irrigation was applied. Sufficient care was paid to the parotid gland and masseteric capsules to complete closure by a resorbable suture, whereas the non-resorbable suture was used for skin closure.

In the SMA group, gentian violet was used to mark the 2–3 cm line below the mandible border. The incision was made and once the exposure was not enough the incision was extended in either direction. Dissection was performed to the platysma muscle, and a blunt scissor was used to bisect the muscle. The cervical fascia was then cut with the care of not causing facial nerve damage. The masseteric sling then incised above the lower border of the mandible, and subperiosteal dissection was achieved until the exposure of the subcondylar area and the reduction and fixation was made. The drilled hole at the angle of the mandible was used to fix the wire used for the reduction.

### Radiological assessment

**Fig. 1:**
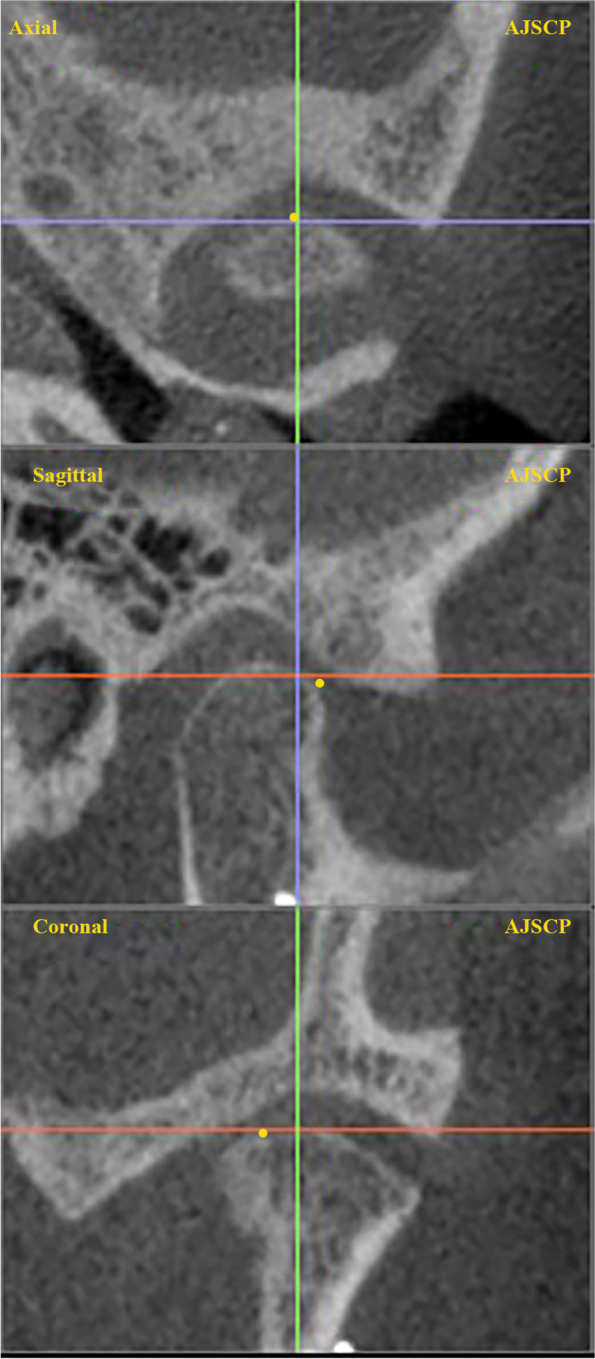
Slice locator position in different planes

Ten days after the operation, a Cone-beam computer tomography was applied to assess the reduction process. All CBCTs were collected spontaneously with patients record. CBCTs were taken in standardized protocol to have the same area of interest without a high discrepancy between patients. CBCTs were collected on DICOM form, and the exposure parameter was set at 20.27 Mas, 120KVP, and 14.9 s. The voxel of the image was also set at 0.4 mm. Three-dimensional analysis was carried out for both groups; RMA and SMA groups. The joint space volume was measured by the equation of sigma V $\cong {\sum }_{k=1}A\left({x}_{1}\right)\Delta \chi$. The whole joint space was sectioned; each section had a width of 1 mm. Tuberculo-metal line (TML), a line from Anterior Tubercle (AT) to Inferior Auditory Meatus (IM) points, was used as the.

inferior border to joint volume. The coordinate system with skeletal midline points was used previously ElBeialy et al., 2011 [14]. Each point was digitized and adjusted by a three-slice locator (Fig. 1).

**Fig. 2:**
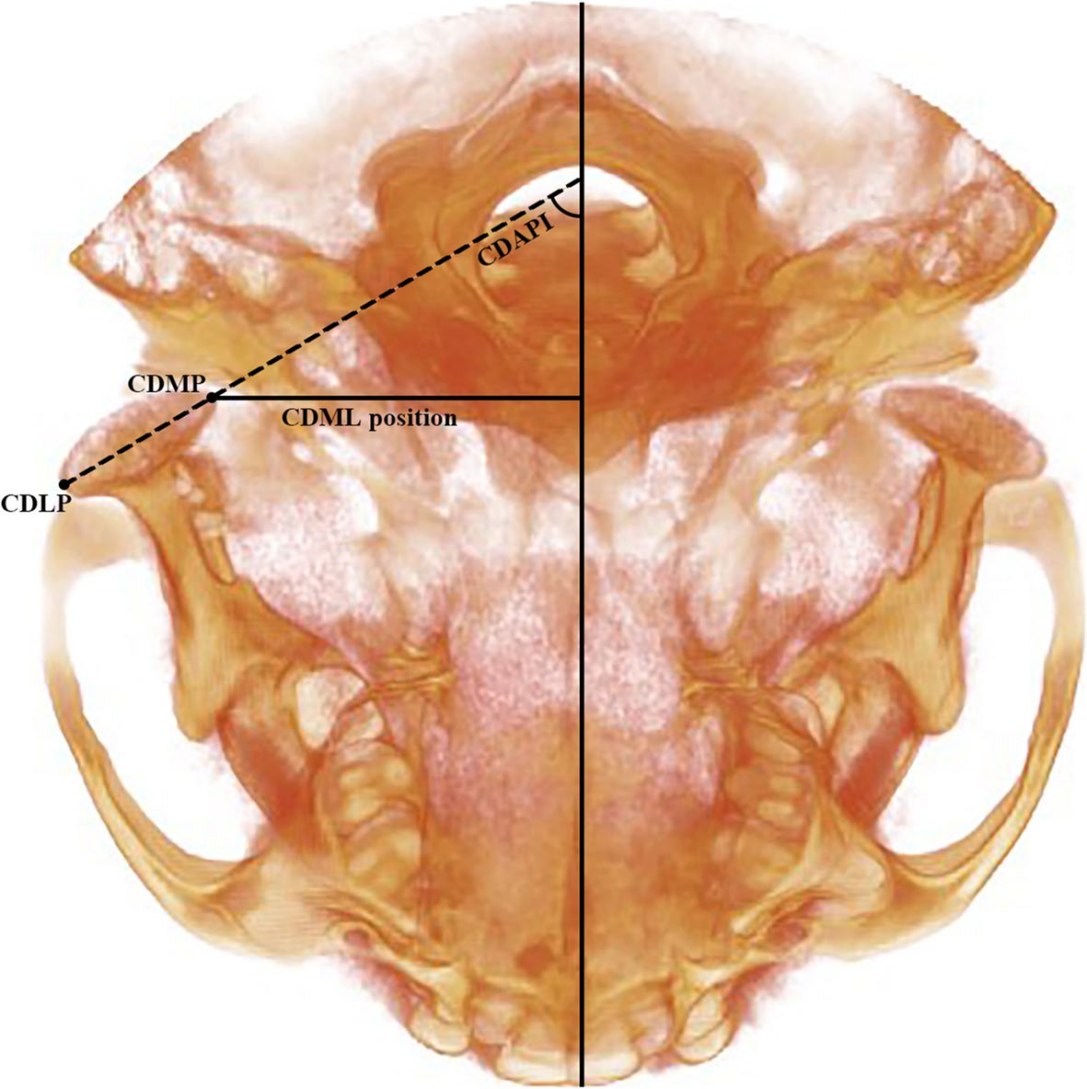
Condylar position and inclination to the midsagittal plane in RMA group. CDMP; condylar medial point, CDLP; condylar lateral point, CDAPi (MSP); condylar anteroposterior inclination to the midsagittal plane, CDML position; condylar mediolateral position

**Fig. 3:**
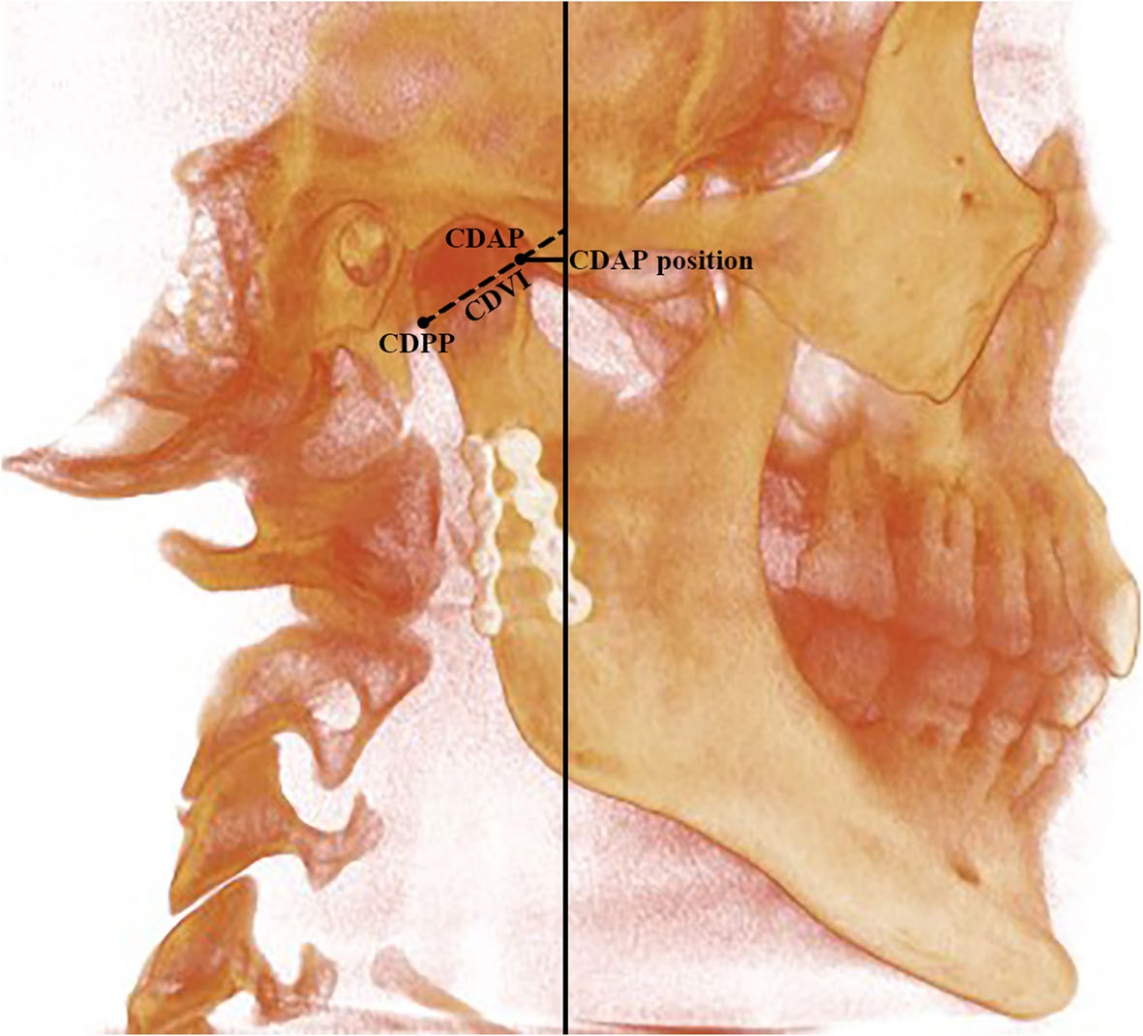
Condylar position and inclination to the vertical plane in RMA group. CDPP; condylar posterior point, CDAP; condylar anterior point, CDVi(VP); condylar vertical inclination to Vertical plane, CDAP position; condylar anterior posterior position

**Fig. 4:**
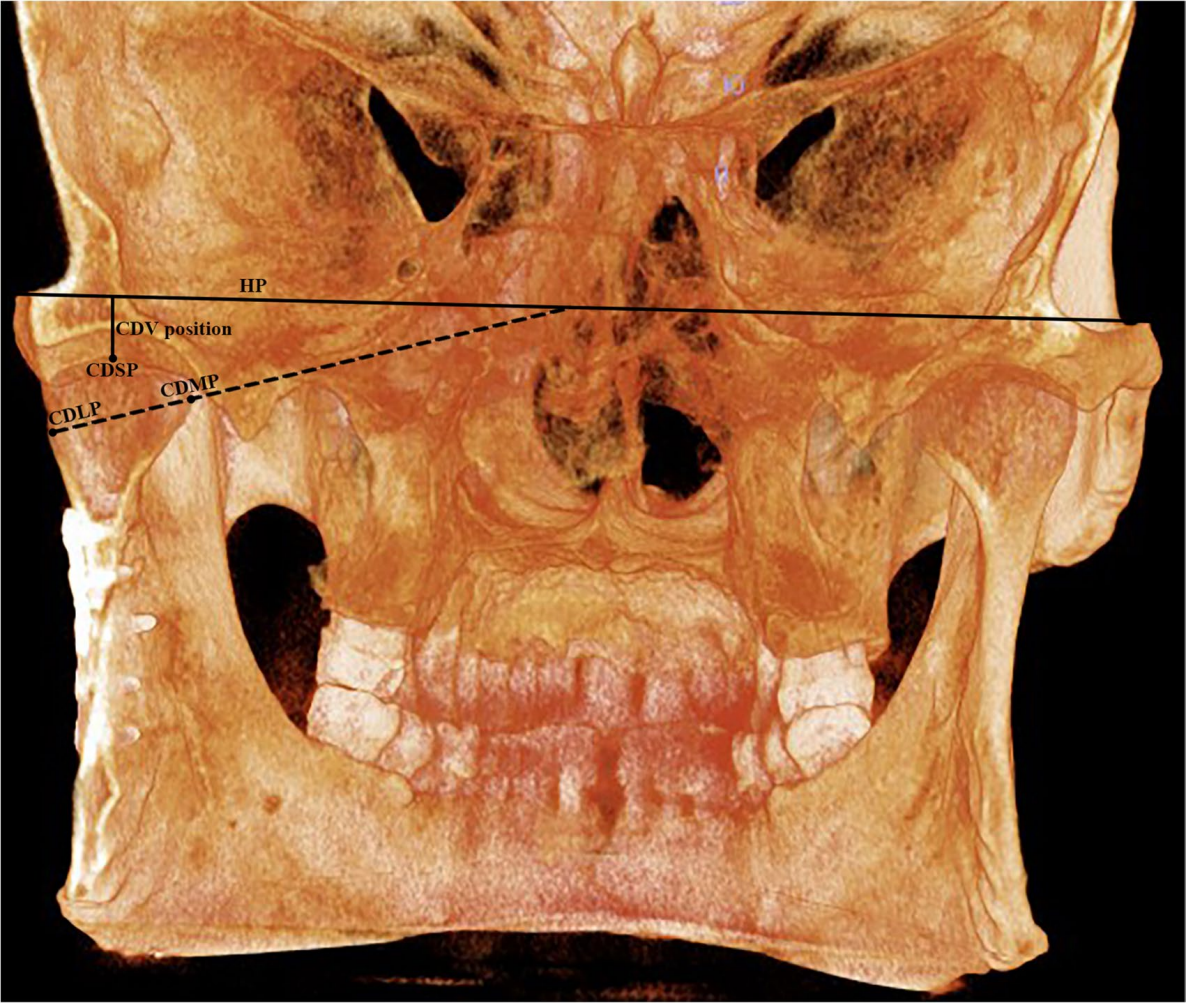
Condylar position and inclination to the horizontal plane in SMA group CDLP; condylar lateral point. CDLP; condylar lateral point, CDMP; condylar medial point, CDSP; condylar superior position, HP; horizontal plane, CDV position; condylar vertical position, CDMLi (HP); condylar mediolateral inclination to horizontal plane

**Fig. 5:**
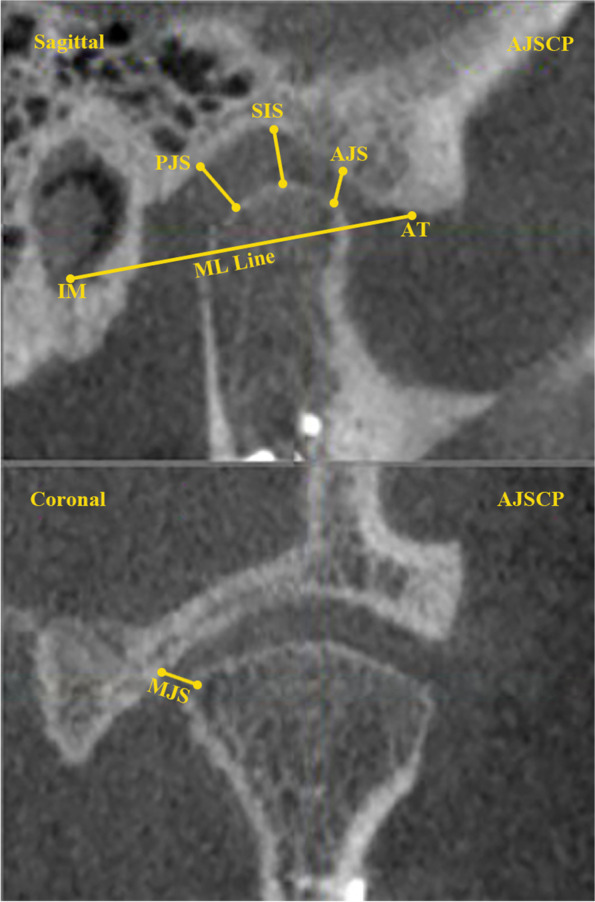
Joint spaces PJS; posterior joint space, SJS; superior joint space, AJS; anterior joint space, MJS; medial joint space, AT; anterior tubercle, IM; auditory meatus, ML line; tuberculo-meatus line

Nasion point was considered the reference point. It was determined on three planes; axial (Y), coronal(X), and sagittal(Z), which were used to build the 3D mold. The three-dimensional equation was used to measure the planes d = $\sqrt{({x}_{1}{-{x}_{2})}^{2}+({y}_{1}-{y}_{2}{)}^{2}+({z}_{1}-{z}_{2}{)}^{2}}$. The Skeletal landmarks, 3-D lines, planes, and measurements for condylar position and angulation are listed in Tables 3 and 4, Figs. 2, 3, 4 and 5 respectively.

National Institutes of Health Database (PubMed) was conducted for the literature review.

### Statistical analysis

Descriptive information was reported as mean and standard deviation. The difference in mean between the two groups was assisted by the independent t-test. Pearson’s correlation analyzed the correlation between the joint space, and condylar position and angulation. Also, the relation between the clinical finding and the fracture side measurement was tested. The interclass correlation was utilized to check the agreement between two independent observers. The *P*-value was set at 0.05 or less to indicate the statistical significance. All analysis was performed by the IBM SPSS Statistics version 26 (IBM Corp., Armonk, NY, USA).

## Results

Thirty-one patients were included in this study; two of them draped off the follow up, and one of them preferred the conservative treatment upon ORIF. Twenty-nine cohort (16 in the RMA group, 13 in the SMA group) with unilateral subcondylar fracture were consecutively included. Demographics and fractures characteristics are shown in Table 1. Of the study population, 23 were male (79.3%), and 6 were female (21.7%). The patient ranged in age from 18 to 69 years, with an average age of 37.2 ± 14.4 years. All patients completed 6 months of follow-up, ranging from 6 to 16 months. Sixteen patients (55.17%) were treated with RMA, and 13 (44.82%) were treated utilizing the SMA. Both groups were treated consecutively by one surgeon. Intra-operative bleeding was minimal, and none of the patients required a blood transfusion. In four cases of the retromandibular group, the retromandibular vein was sectioned and retracted posteriorly. Operative time in retromandibular patients was shorter than submandibular patients, but the difference was not significant. The parotid fistula was not detected in any case. The facial nerve was encountered in both groups, and temporary weakness was seen in three patients. No permanent facial nerve weakness was detected at the end of the follow-up time. Postoperative malocclusion was found in two patients and was treated by elastic traction. Frey’s syndrome, wound infection, abscess, pus discharge, or cellulitis were not detected. Concomitant fractures were treated with a suitable osteosynthesis set.

Helkimo index was conducted to evaluate the patients. In patients with retromandibular approach, AiO was found in 12 patients, whereas the AiI was found in 4 patients. However, the objective clinical finding DiO, I, II were found in 2,10 and 4, respectively. Patients treated with submandibular approach had AiO in 7 patients, while the DiI was found in 5 patients (Table 5).

### Radiological assessment

The measurement and parameter between the mean of the fracture and non-fracture side for each approach were tested with an independent *t*-test and listed in Tables 6 and 7.

The intercorrelation coefficient between the two independent observers was 8.5, indicating that excellent reliability.

On RMA group, the horizontal, vertical, and midsagittal condylar angulation on the fracture side were (10.7 ± 2°), (61.7 ± 19°), and (72 ± 7°), whereas on the patients with SMA fracture side were (8.4 ± 2°), (57.8 ± 11°), and (64.2 ± 9°), respectively. However, the condylar position to the horizontal plane on RMA was (2.1 ± 0.7 mm) and on SMA patients was (3.3 ± 0.7 mm). The condylar position to the vertical plane in RMA’s fracture side was (7.7 ± 4 mm); however, on SMA was (7.6 ± 1.7 mm). In addition, the condylar position to the midsagittal plane on RMA was 52.9 ± 4 mm and on SMA was 46 ± 3.6 mm.

The mediolateral condylar inclination to the horizontal plane on SMA was significantly lower than with RMA (*P* = 0.02). Furthermore, the anteroposterior condyle inclination to the midsagittal plane was lower on SMA than on the RMA group (*P* = 0.01). The mediolateral condyle position was higher on the RMA than SMA (*P* = 0.001).

Regarding the joint space, the differences between the fracture sides of the SMA and RMA was tested, the superior, medial, anterior, and posterior joint spaces on SMA patients were 3.2 ± 1 mm, 1.8 ± 0.7 mm, 2.5 ± 0.7 mm, and 1.9 ± 0.6 mm, whereas on RMA’s fracture side were 2.3 ± 0.7 mm, 2.6 ± 1 mm, 2.7 ± 0.6 mm and 2.2 ± 0.4 mm.

On the other hand, the joint spaces were tested to find the correlation between condylar position and inclination. The medial joint space was positively related to the condylar mediolateral position in both approaches (*P* < 0.05). However, a positive relation was detected on SMA between the anterior joint space and the anteroposterior condylar position (*P* = 0.008). Furthermore, the superior joint space had a positive relationship with the vertical condylar position on SMA patients (P = 0.004; Table 8).

In comparing the 3D finding and the Helkimo index. A negative relationship was identified between the mediolateral condylar angulation and Helkimo Ai (subjective) and Helkimo Di (objective) on SMA (*P* < 0.05). However, on RMA, the same condylar angulation was significantly related with Helkimo Ai. Furthermore, the mediolateral condylar position had a negative relationship with the Helkimo Ai on the submandibular approach (Table 9).

## Discussion

For the subcondylar fracture, the retromandibular approach provides better exposure to the subcondylar fracture region than the other approaches. However, this approach encounters the facial nerve branches; the buccal and marginal mandibular branches. Despite the meticulous identification of the facial nerve, this method necessitates parotid gland retraction, which may result in facial nerve injury [15–18]. Although the RMA provides ample exposure to the surgical site, the reduction along the buccal surface does not assure a 3-D reduction [19]. On the other hand, the subcondylar fracture line will be approached by the incision below the marginal mandible nerve branch on the submandibular approach. The marginal mandibular nerve is easily retracted within the superior layer of the deep cervical facia. In contrast, other approaches need to identify the facial nerve, which is difficult for inexperienced surgeons. The submandibular approach is straightforward to perform and does not need a virtual learning curve [10]. However, this approach provides less exposure to the submandibular surgical side through the sufficient detachment of the masseter muscle from the posterior border of the mandibular ramus [10].

Regarding the condylar inclination in both groups, we found the mediolateral condylar inclination to the horizontal plane was significantly lower on the submandibular approach, confirming that the medial inclination of the proximal part, which it is similar to the finding of other studies [20, 21]. The anteroposterior condylar inclination to the midsagittal plane was lower than the retromandibular approach. This result is opposite to what Choi et al. found. However, it is in line with previous study [12]. Interestingly, the mediolateral inclination of the condyle to the horizontal plane was significantly correlated, in submandibular approach patients, with both Helkimo Ai and Helkimo Di. This finding indicates the relationship between the mediolateral condyle rotation with the clinical objective and subjective outcome, which is in line with who stated that the rotation of the condyle could be associated with popping sound [22].

The condyle position to the midsagittal plane was on submandibular patients smaller than on the retromandibular patients *P* = 0.02, which indicates that the fracture stump is located more medially. Additionally, there was a positive relationship between the medial joint space and medial condylar position. This finding is similar to Hlawitschka et al., who stated that the postsurgical condyle displacement was medial [23]. Briefly, we think that the superior head of the lateral pterygoid muscle and the insufficient exposure of the surgical site can lead to this finding.

In comparing the joint spaces in submandibular and retromandibular approaches, the medial joint space on submandibular patients was lower than the retromandibular patients, which correlated with mediolateral condylar positional change. However, the other joint spaces were not significantly different, and there was no correlationship between the change in joint space with neither Helkimo Hi nor Helkimo Di.

Although the panoramic radiography is the most popular in dental practice and is frequently used to evaluate the fracture after surgical operation, the CBCTs examination for the condylar fracture provides a three-dimensional view and delineates any fine condylar changes without superimposition nor distortion. Furthermore, the CBCT was found to be more accurate than other methods in determining the condyle points [24, 25]. For that, CBCT was used in our study to evaluate the condylar anatomical position.

There are many different evaluation systems to assess the functional impairment of the TMJ, for example, the Mandibular Function Impairment Questionnaire, Craniomandibular Index, and Helkimo Index [26, 27]. Helkimo Index has been used to assess the function of the TMJ after the open reduction and internal fixation of the mandibular condylar fracture [23, 28]. Furthermore, studies used Helkimo Index to assess the relationship between malocclusion, TMD, and muscle activity [29, 30]. Kordass et al. concluded a significant relationship between the popping sound and Helkimo’s Di during the mandibular movement [22]. In our study, the Helkimo index was applied as it is reliable and easy to use [31, 32].

RMA and SMA provide good access to the subcondylar fracture; however, patients’ images showed more accurate redaction of the condyle position and angulation in the retromandibular approach. Although the direct access into the surgical field and buccal surface help the surgeon to apply more anatomical reduction and fixation, the complete three-dimensional reduction is still not fully re-established [33]. Moreover, the mediolateral condyle inclination in SMA was related to subjective and objective clinical symptoms, which indicates the rotation of the condyle with the horizontal plane (the path of condylar movement) can be connected to clinical outcome.

The strength of this study was the nature of prospective design and the use of the CBCT to systematically evaluate the accuracy of condylar reduction and compare it with the Helkimo index of the TMJ functional impairment at a six-month follow-up. This can give the surgeon a thought to anticipate the patient outcome related to the CBCT after surgical operation. Furthermore, this study was the first to use three-dimensional CBCT to compare the SMA with RMA concerning the reduction accuracy. This study has limitations. The relatively small sample size in population, and the inhomogeneous distribution of the fracture among the groups might make this study prone to bias. Furthermore, the follow-up time was relatively short, and the CBCT was only taken ten days postoperatively.

## Conclusion

The SMA and RMA were systematical radiographically evaluated. The RMA was more able to re-establish the three-dimensional reduction of the subcondylar fracture. In contrast, the SMA was related with decreasing in the mediolateral condylar inclination, which is related to the Helkimo Ai and Helkimo Di outcome.

**Table 1:** Demographic data for patients

	Retromandibular	Submandibular
Number	16	13
Gender		
Male	12	11
Female	4	2
Fracture side		
Right	10	6
Left	6	7
Cause of fracture		
Fall down	11	10
Traffic accident	5	2
Fighting	0	1
Concomitant Fracture		
symphysis	10	9
Contralateral body fracture	3	1
No other fracture	2	2
Other	1	1
Interincisal opening (mean)	42.35	40.23
Time of operation minutes (mean)	80	100
Temporary facial nerve weakness (No.)	2	1
Permanent facial nerve weakness	0	0

**Table 2:** Helkimo Ai and Di index score

Subjective Helkimo Ai Index		
Subjective Helkimo index	AiO	Asymptomatic; no symptoms reported
	AiI	Mild symptomatic; One of these was found; stiffness in the morning, and noise of the joint, fatigue in mastication of muscle
	AiII	Severe symptomatic; Mouth movement limitation, joint locking or dislocation, pain during the mouth movement and or muscle of mastication
Objective Helkimo Di Index		
Range of mandibular movement	Normal	0
	Relatively impaired	1
	Impaired	5
Temporomandibular function	Sound and deviation un reported	0
	Sound or deviation more than 2 mm	1
	Locking or luxation	5
Muscles pain	No tenderness	0
	Tenderness with palpation (from 1 to 3 sites)	1
	Tenderness with palpation (at least 4 sites)	5
TMJ pain	No tenderness	0
	Tenderness during laterally palpation	1
	Tenderness during posterior palpation	5
Pain on mandibular movement	No pain	0
	Pain on one movement	1
	Pain on two or more movements	5

*DiO *  0, *DiI*   1to 4, *DiII*  5 to 9, *DiIII*   10 to 30

**Table 3:** Landmarks Definition

Nasion (N)	In the midline of Nasofrontal suture
Medial joint space (MJSF) “mandibular fossa point”	The most lateral point of the medial wall of mandibular fossa
Condyle Superior point (CDSP)	The most superior midpoint of the condylar head
Condyle Medial point (CDMP)	The Most lateral inner wall point of the condyle head
Condyle Lateral point (CDLP)	The most oblique point in the lateral side of condyle head
Condyle Anterior point (CDAP)	The most anterior point of the condylar head
Condyle Posterior point (CDPP)	The most posterior point of the condylar head
Inferior meatus (IM)	The most inferior and lateral point of external auditory meatus
Articular Tubercle (AT)	The most inferior posterior point of the articular tubercle
Anterior joint-space Mandibular fossa (AJSF)	The most posterior point of the anterior wall of the mandibular fossa opposed to the shortest distance of anterior condylar-fossa
Anterior joint-space Condylar point (AJSc)	The most anterior point of the condyle head opposed to the shortest distance of anterior condylar-fossa
Posterior joint-space Mandibular fossa (PJSf)	The most anterior point of the posterior wall of the mandibular fossa opposed to the shortest distance of posterior condylar-fossa
Posterior joint-space Condylar point (PJSc)	The most posterior point of the condyle opposed to the shortest distance of posterior condylar-fossa
Medial joint-space Mandibular fossa (MJSf)	The most lateral point of the inner medial wall of mandibular fossa opposed to the shortest distance of medial fossa-condyle
Medial joint-space Condylar point (MJSc)	The most lateral point of the medial condylar head opposed to the shortest distance of medial fossa-condyle

**Table 4:** Fracture and non-fracture sides for each approach

Independent *t*-test										
Acronym	Submandibular approach				*P *value	Retromandibular approach				*P *value
	Fracture side		Non-fracture side			Fracture side		Non-fracture side		
	Mean	SD	Mean	SD		Mean	SD	Mean	SD	
CDMLi (HP)	8.4	2	13.3	3	**0.001**	10.7	2	12.2	1.7	**0.03**
CDVi (VP)	57.8	11	60.9	8	0.45	61.7	19	66.6	11.2	0.4
CDAPi (MSP)	64.2	9	76.8	5	**0.001**	72	7	74	6.8	0.42
CDVp	3.3	0.7	2.5	0.6	**0.007**	2.1	0.7	2.7	0.7	**0.01**
CDAPp	7.6	1.7	6.8	3	0.38	7.7	4	7.3	2.5	0.74
CDMLp	46	3.6	49.9	4	**0.02**	52.9	4	50.2	3.3	0.06
AJS	2.5	0.7	2.1	0.7	0.11	2.7	0.6	2.1	0.8	**0.03**
PJS	2.1	0.4	2.4	0.2	0.06	2.2	0.4	2.1	0.4	0.71
MJS	1.8	0.7	2.6	1	**0.04**	2.6	1	2.8	1.2	0.57
SJS	3.2	1.1	2.2	0.7	0.21	2.3	0.7	2	0.6	0.25

Bold is less than 0.05

**Table 5:** Helkimo index result in fractured patients

Approach	Retromandibular	Submandibular
Ai		
AiO	12	7
AiI	4	5
AiII	0	1
Di		
DiO	2	1
DiI	10	6
DiII	4	5
DiIII	0	1

**Table 6:** Fracture and non-fracture sides for each approach

Independent* t*-test										
Acronym	Submandibular approach				*P *value	Retromandibular approach				*P *value
	Fracture side		Non-fracture side			Fracture side		Non-fracture side		
	Mean	SD	Mean	SD		Mean	SD	Mean	SD	
CDMLi (HP)	8.4	2	13.3	3	**0.001**	10.7	2	12.2	1.7	**0.03**
CDVi (VP)	57.8	11	60.9	8	0.45	61.7	19	66.6	11.2	0.4
CDAPi (MSP)	64.2	9	76.8	5	**0.001**	72	7	74	6.8	0.42
CDVp	3.3	0.7	2.5	0.6	**0.007**	2.1	0.7	2.7	0.7	**0.01**
CDAPp	7.6	1.7	6.8	3	0.38	7.7	4	7.3	2.5	0.74
CDMLp	46	3.6	49.9	4	**0.02**	52.9	4	50.2	3.3	0.06
AJS	2.5	0.7	2.1	0.7	0.11	2.7	0.6	2.1	0.8	**0.03**
PJS	2.1	0.4	2.4	0.2	0.06	2.2	0.4	2.1	0.4	0.71
MJS	1.8	0.7	2.6	1	**0.04**	2.6	1	2.8	1.2	0.57
SJS	3.2	1.1	2.2	0.7	0.21	2.3	0.7	2	0.6	0.25

Bold is less than 0.05

**Table 7:** Fracture sides in both approaches was tested by independent t-test

Approach	Submandibular approach Fracture aide		Retromandibular approach Fracture side		*P* value
Acronym	Mean	SD	Mean	SD	
CDMi (HP)	8.4	2	10.7	2	**0.02**
CDVi (VP)	57.8	11	61.7	19	0.53
CDAPi (MSP)	64.2	9	72	7	**0.01**
CDVp	3.3	0.7	2.1	0.7	**0.001**
CDAPp	7.6	1	7.7	4	0.98
CDMp	46	3	52.9	4	**0.001**
SJS	3.2	1	2.3	0.7	**0.03**
MJS	1.8	0.7	2.6	1	**0.03**
AJS	2.5	0.7	2.7	0.6	0.58
PJS	1.9	0.6	2.2	0.4	0.19

Bold is less than 0.05

**Table 8:** Joint spaces with different parameters in each approach

Submandibular approach								
Acronym	AJS		PJS		MJS		SJS	
	Pearson	*P* value	Pearson	*P* value	Pearson	*P* value	Pearson	*P* value
CDMLi (HP)	.142	.643	-.379	.202	-.104	.73	-.211	.489
CDVi (VP)	.546	.054	.216	.479	-.005	.987	.060	.845
CDAPi (MSP)	-.106	.729	-.280	.354	-.231	.447	.067	.828
CDVp	.226	.458	-.068	.824	-.009	.976	.739	**.004**
CDAPp	.699	**.008**	-.085	.782	-.320	.287	.095	.757
CDMLp	.537	.059	.458	.458	.571	**.041**	.142	.644
Retromandibular approach								
CDMLi (HP)	.265	.322	.402	.123	.267	.317	.250	.349
CDVi (VP)	.223	.407	.136	.615	-.596	**.015**	-.535	**.033**
CDAPi (MSP)	-.168	.534	-.040	.884	-.303	.254	.303	.255
CDVp	-.195	.469	-.332	.209	-.303	.255	.275	.303
CDAPp	-.100	.710	-.153	.570	-.111	.683	.291	.274
CDMLp	-.247	.357	-.496	.051	.616	**.011**	.065	.812

Bold is less than 0.05

**Table 9:** Correlation with clinical outcome

Approach	Submandibular				Retromandibular			
Acronym	Helkimo Ai		Helkimo Di		Helkimo Ai		Helkimo Di	
	Pearson test	*P* value	Pearson test	*P* value	Pearson test	*P* value	Pearson test	*P* value
CDMLi (HP)	-.579	**.038**	-.638	**.019**	-.704	**.002**	-.480	.060
CDVi (VP)	-.437	.106	-.364	.221	.219	.414	.400	.125
CDAPi (MSP)	-.293	.332	-.651	**.016**	-.015	.957	-.011	.968
CDVp	-.326	.276	-.410	.164	-.278	.298	.683	**.004**
CDAPp	-.056	.855	.034	.913	.036	.895	-.018	.948
CDMlp	-.611	**.026**	.235	.440	.131	.628	.019	.944
AJS	-.111	.717	.083	.787	-.159	.556	.046	.864
PJS	-.112	.714	-.378	.203	-.331	.210	-.509	**.044**
MJS	.117	.704	.190	.534	-.526	**.036**	-.105	.699
SJS	-.191	.532	-.167	.586	-.290	.277	-.117	.665

Bold is less than 0.05

## Supplementary Information

**Additional file 1.**

## Data Availability

The datasets used and/or analyzed during this study are available from the corresponding author on reasonable request.
