# Supplementary material for: Is the retromandibular approach a suitable approach to anatomical reduction of unilateral subcondylar fracture? A non-randomized clinical trial
Source: Head Face Med. 2022 Dec 2;18:38. doi: 10.1186/s13005-022-00342-1 (PMC9716850; doi:10.1186/s13005-022-00342-1)
Supplement: Supplementary file 1 — Additional file 1. [file 13005_2022_342_MOESM1_ESM.pdf]

Article type: original search

Is the Retromandibular approach a suitable approach to anatomical reduction of unilateral subcondylar fracture? A non-randomized clinical trial

Abdo Ahmed Saleh Mohamed[1,2,3]†, Rao Guangxin[1]†, Mai Lianxi[1], Karim Ahmed Sakran[2,4], Saddam Noman Al-wesabi[5], Chaobin Pan[1]* Zhaoyu Lin[1]**

[1] Department of Oral & Maxillofacial Surgery, Sun Yat-sen Memorial Hospital, Sun Yat-sen University, Guangzhou, China
[2] Department of Oral and Maxillofacial Surgery, Faculty of Dentistry, Ibb University, Ibb, Yemen

[3] Department Oral and Maxillofacial Surgery, Lanzhou University First Affiliated Hospital, Lanzhou University, Lanzhou, Gansu Province, China

[4] Resident, State Key Laboratory of Oral Diseases and National Clinical Research Center for Oral Diseases and Department of Oral and Maxillofacial Surgery, West China Hospital of Stomatology, Sichuan University, Chengdu, China Assistant
[5] Department of Oral and Maxillofacial surgery, School of stomatology, Lanzhou University, Lanzhou, Gansu, China

[*] Correspondence to professor Chaobin Pan, Department of Oral & Maxillofacial Surgery, Sun Yat-sen Memorial Hospital, Sun Yat-sen University, Guangzhou, China
** Co-corresponding author
† The authors equally contributed
Email:
Abdomohamed4010@icloud.com
chaobinpan22@gmail.com

Abstract

The aim of this study was to evaluate the efficacy of the retromandibular approach (RMA) to produce three-dimensional (3-D) reduction of the unilateral subcondylar fracture and Temporomandibular Joint (TMJ) functional implication.

Methods: A prospective cohort study was designed. Twenty-nine patients with unilateral subcondylar fracture underwent consecutively Open Reduction, and Internal Fixation. The cohorts were divided into two groups; RMA group (n=16, 55.17%) and submandibular approach SMA group (n= 13, 44.82%). The primary outcome was the anatomical 3-D reduction of the condyle. The secondary outcome was to compare the condyle position and inclination finding with TMJ outcomes. Helkimo Index score was used to evaluate the TMJ outcome at six months postoperatively.

Result: There was a significant difference between the mediolateral condylar inclination, condylar medial and vertical positions when RMA compared with SMA groups ($P < 0.05$). The medial joint space was correlated with the medial condylar position in both groups ($P < 0.05$). The Helkimo Ai and Di was associated with mediolateral condylar inclination in SMG; however, Helkimo Ai was found to be correlated with the RMA group.

Conclusion: The current study demonstrates that the RMA could re-establish the anatomical position of the unilateral subcondylar fracture in patients undergoing ORIF. The clinical outcome of the TMJ with RMA was better than SMA

Keywords: Condylar fracture, Retromandibular approach, Reduction accuracy, Three-dimensional CBCT analysis.

Background

Condyle fracture is one of the most fractures in the mandibular bone. It ranges from 25% to 50% of the mandibular fracture.(1,2) The location and the direction of the fracture line might determine the classification of the fracture. In general, condyle fracture has been classified based on the anatomical location of the fracture line into three types; condyle head, condyle neck, and condyle base (subcondylar) fractures.(3) The treatment for condyle fracture varies from the closed condyle treatment (CCT) to the open reduction and internal fixation (ORIF). Each approach has its own set of pros and cons; however, this is still a topic of controversy.(2,4) The most hazardous effect with the open approach is the facial nerve injury, whereas in the closed approach, is the ability to reduce the condyle to its normal position. A fracture line that starts above the sigmoid notch and, 2/3 of it, passes down the sigmoid notch is called subcondylar fracture.

The treatment of the subcondylar fracture has been debated for a long time; however, recently, there has been a trend to be treated by open reduction and internal fixation.(5,6) ORIF is performed to get direct access to the fracture line. Therefore, many surgical approaches have been developed to manage the subcondylar fracture, for instance, submandibular, high submandibular, retromandibular, and intraoral (with or without endoscopic/transbuccal trocar) approaches. Although these approaches have the risk of jeopardizing the facial nerve function and other postsurgical complications, the high submandibular approach (HSMA) or retromandibular approach (RMA) were the safest approaches concerning minimizing the facial nerve injury.(5)

Furthermore, the anatomical location and proximity of the subcondylar fracture to the TMJ can have a long-term functional impairment, especially if proper anatomical relationships are not accurately re-established. Generally, anatomical reduction and stability of subcondylar fracture after ORIF are multifactorial issue, affected not only by the approach but also by the level of the fracture line, number and type of bone plates and also whether it is 2-D or 3-D plate. Plus, concomitant fractures and the way to fix it.(7,8) Huang CM et, al. reported that placing the miniplates fixation in the posterior margin of the ascending ramus margin increases the fixation stability (9)

Commented [MOU1]: References added

For that, providing an excellent surgical field is very important to have successful reduction. RMA and SMA approaches have been advocated to expose, operatively reduce, and fix these fractures. Previous studies concluded that both methods were able to provide reasonable access and comparable postoperative clinical results.(10,11) Radiographically, few studies analyze the condyle position after ORIF. Our previous study has highlighted the condylar positional change with ORIF regardless of the surgical incision approach.(12) To the best of our knowledge, no paper discussed the condyle position, angulation, and joint space - anatomical reduction- between the RMA and the SMA for the subcondylar fracture. We hypothesized that the reduction of the condyle and the clinical outcome are not significantly different either by the RMA or SMA. This article aimed to answer the following questions:

1- Does the Retromandibular approach provide anatomical reduction for the unilateral condyle fracture over the submandibular approach?
2- Do the clinical outcomes differ with different approaches ?

This article used the CBCT to provide a detailed information related to the anatomical reduction of the subcondylar fracture and its clinical outcome in temporomandibular joint.

Methods

Study design

This prospective non-randomized cohort study was conducted at the 1st Hospital of Lanzhou University, Department of Oral and Maxillofacial Surgery, from September 2017 to February 2020. Twenty-nine consecutive patients who underwent ORIF (RMA or SMA) for the unilateral subcondylar fracture were included. All patients were evaluated presurgical with clinical examination and Panoramic/CT-scan. An informed consent form, including explanation about the two approaches, was obtained from patients. The cohorts were informed of the right to refuse to participate in the study or to withdraw consent to participate at any time without reprisal

This study followed the Declaration of Helsinki on medical protocol and ethics and the regional Ethical Review Board of Stomatology College, Lanzhou University approved the study. The postoperative 3-D CBCTs were used for radiological evaluation. The clinical assessment was conducted by malocclusion, mouth opening limitation, and postoperative pain.

Comparing between retromandibular and submandibular approaches was implemented. Also, the fracture side and non-fracture side were analyzed.

The patients included in this study were suffering from simple unliteral subcondylar fracture, with 5° to 40° deviation between the subcondylar and the ascending ramus, more than 2 mm shortage of ramus, older than 18 years old, and limitation of mouth opening. Patients with any history of TMD, less than 18 years old, bilateral subcondylar fracture, condylar head fracture, insufficient dentition, treated with closed treatment or endoscopic and patients with comminuted fractures were excluded from this study. Demographic data is included in table 1.

Table 1. Demographic data for patients

	Retromandibular	Submandibular
Number	16	13
Gender		
Male	12	11
Female	4	2
Fracture side		
Right	10	6
Left	6	7
Cause of fracture		
Fall down	11	10

Traffic accident	5	2
Fighting	0	1
Concomitant Fracture		
symphysis	10	9
Contralateral body fracture	3	1
No other fracture	2	2
Other	1	1
Interincisal opening (mean)	42.35	40.23
Time of operation minutes (mean)	80	100
Temporary facial nerve weakness (No.)	2	1
Permanent facial nerve weakness	0	0

The Comprehensive AOCMF Classification System by Neff, A., et al., 2014 was used in this study.(13)

Clinical assessment

Helkimo index scoring system was implemented in the current study to evaluate TMJ function. Helkimo Ai has utilized the TMJ dysfunction subjectively. On the other hand, Di represented the objective assessment of the impaired TMJ function. Both subjective and objective symptoms were evaluated by the limitation of mouth opening, TMJ function impairment, pain in the muscle, and TMJ. The patient was classified as Ai0 (asymptomatic), AII (Mild symptoms), AiIII (severe symptoms). The score for the Di is shown in table 2.

Table 2 Helkimo Ai and Di index score		
	Subjective Helkimo Ai Index	
Subjective Helkimo index	AiO	Asymptomatic; no symptoms reported
	AiI	Mild symptomatic; One of these was found; stiffness in the morning, and noise of the joint, fatigue in mastication of muscle
	AiII	Severe symptomatic; Mouth movement limitation, joint locking or dislocation, pain during the mouth movement and or muscle of mastication
	Objective Helkimo Di Index	
Range of mandibular movement		
	Normal	0
	Relatively impaired	1

	Impaired	5
Temporomandibular function		
	Sound and deviation un reported	0
	Sound or deviation more than 2mm	1
	Locking or luxation	5
Muscles pain		
	No tenderness	0
	Tenderness with palpation (from 1 to 3 sites)	1
	Tenderness with palpation (at least 4 sites)	5
TMJ pain		
	No tenderness	0
	Tenderness during laterally palpation	1
	Tenderness during posterior palpation	5
Pain on mandibular movement		
	No pain	0
	Pain on one movement	1
	Pain on two or more movements	5
	DiO= 0 , DiI= 1to 4, DiII= 5 to 9 , DiIII= 10 to 30	

Patients were followed for six months (at least). Pain in the temporomandibular joint (TMJ) region, facial nerve weakness, occlusion disturbances, and interincisal mouth opening were assessed.

Surgical operation

All the Open Reduction and Internal Fixation was performed by one surgeon in consecutive pattern. Patients underwent ORIF under general anesthesia. The RMA and SMA procedures are outlined below:

In the RMA group, it was similar to what Ellis and Dean described. Shortly, gentian violet was used to mark the subcutaneous skin incision (3-4 cm) below the ear pinna and 1 cm behind the angle of the mandible. Dissection was made until the subplatysmal layer. Once the parotid gland fascia was identified, the blind dissection was carried out parallel with facial nerve direction; once facial nerves were encountered, they were first carefully dissected and retracted to decrease the tension. The periosteum was incised at the posterior border of the mandibular. After the subperiosteal dissection of the ramus and subcondylar region, the reduction and fixation of the fracture fragments were obtained. For providing enough working space on the fracture line, the manual downward pressure was applied. Two suitable 2.0 mm miniplates were used to fix the fracture sides, and then copious irrigation was applied. Sufficient care was paid to the parotid gland and masseteric capsules to complete closure by a resorbable suture, whereas the non-resorbable suture was used for skin closure.

In the SMA group, gentian violet was used to mark the 2-3 cm line below the mandible border. The incision was made and once the exposure was not enough the incision was extended in either direction. Dissection was performed to the platysma muscle, and a blunt scissor was used to bisect the muscle. The cervical fascia was then cut with the care of not causing facial nerve damage. The masseteric sling then incised above the lower

border of the mandible, and subperiosteal dissection was achieved until the exposure of the subcondylar area and the reduction and fixation was made. The drilled hole at the angle of the mandible was used to fix the wire used for the reduction.

Radiological assessment

Ten days after the operation, a Cone-beam computer tomography was applied to assess the reduction process. All CBCTs were collected spontaneously with patients record. CBCTs were taken in standardized protocol to have the same area of interest without a high discrepancy between patients. CBCTs were collected on DICOM form, and the exposure parameter was set at 20.27 Mas, 120KVP, and 14.9s. The voxel of the image was also set at 0.4 mm. Three-dimensional analysis was carried out for both groups; RMA and SMA groups. The joint space volume was measured by the equation of sigma $V \cong \sum_{k=1} A\,(x_1)\Delta\chi$. The whole joint space was sectioned; each section had a width of 1 mm. Tuberculo-metal line (TML), a line from Anterior Tubercle (AT) to Inferior Auditory Meatus (IM) points, was used as the

inferior border to joint volume. The coordinate system with skeletal midline points was used previously ElBeialy et al., 2011.(14) Each point was digitized and adjusted by a three-slice locator (Figure 1).

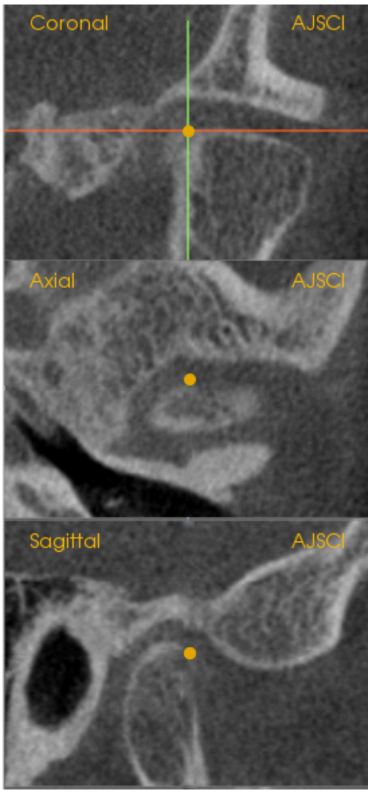

Nasion point was considered the reference point. It was determined on three planes; axial (Y), coronal(X), and sagittal(Z), which were used to build the 3D mold. The three-dimensional equation was used to measure the planes d=$\sqrt{(x_1-x_2)^2+(y_1-y_2)^2+(z_1-z_2)^2}$. The Skeletal landmarks, 3-D lines, planes, and measurements for condylar position and angulation are listed in table 3,4, figures 2, 3, 4 respectively.

Table 3. Landmarks Definition	
Nasion (N)	In the midline of Nasofrontal suture
Medial joint space (MJSF) “mandibular fossa point”	The most lateral point of the medial wall of mandibular fossa
Condyle Superior point (CDSP)	The most superior midpoint of the condylar head
Condyle Medial point (CDMP)	The Most lateral inner wall point of the condyle head
Condyle Lateral point (CDLP)	The most oblique point in the lateral side of condyle head
Condyle Anterior point (CDAP)	The most anterior point of the condylar head
Condyle Posterior point (CDPP)	The most posterior point of the condylar head
Inferior meatus (IM)	The most inferior and lateral point of external auditory meatus
Articular Tubercle (AT)	The most inferior posterior point of the articular tubercle
Anterior joint-space Mandibular fossa (AJSF)	The most posterior point of the anterior wall of the mandibular fossa opposed to the shortest distance of anterior condylar-fossa
Anterior joint-space Condylar point (AJSc)	The most anterior point of the condyle head opposed to the shortest distance of anterior condylar-fossa
Posterior joint-space Mandibular fossa (PJSf)	The most anterior point of the posterior wall of the mandibular fossa opposed to the shortest distance of posterior condylar-fossa
Posterior joint-space Condylar point (PJSc)	The most posterior point of the condyle opposed to the shortest distance of posterior condylar-fossa
Medial joint-space Mandibular fossa (MJSf)	The most lateral point of the inner medial wall of mandibular fossa opposed to the shortest distance of medial fossa-condyle
Medial joint-space Condylar point (MJSc)	The most lateral point of the medial condylar head opposed to the shortest distance of medial fossa-condyle

Table 4, Fracture and non-fracture sides for each approach										
Independent *t*-test										
Acronym	Submandibular approach				P value	Retromandibular approach				P value
	Fracture side		Non-fracture side			Fracture side		Non-fracture side		
	Mean	SD	Mean	SD		Mean	SD	Mean	SD	
CDMLi (HP)	8.4	2	13.3	3	**0.001**	10.7	2	12.2	1.7	**0.03**
CDVi (VP)	57.8	11	60.9	8	0.45	61.7	19	66.6	11.2	0.4
CDAPi (MSP)	64.2	9	76.8	5	**0.001**	72	7	74	6.8	0.42
CDVp	3.3	0.7	2.5	0.6	**0.007**	2.1	0.7	2.7	0.7	**0.01**
CDAPp	7.6	1.7	6.8	3	0.38	7.7	4	7.3	2.5	0.74
CDMLp	46	3.6	49.9	4	**0.02**	52.9	4	50.2	3.3	0.06
AJS	2.5	0.7	2.1	0.7	0.11	2.7	0.6	2.1	0.8	**0.03**
PJS	2.1	0.4	2.4	0.2	0.06	2.2	0.4	2.1	0.4	0.71
MJS	1.8	0.7	2.6	1	**0.04**	2.6	1	2.8	1.2	0.57
SJS	3.2	1.1	2.2	0.7	0.21	2.3	0.7	2	0.6	0.25

Bold is less than 0.05

Figure 2

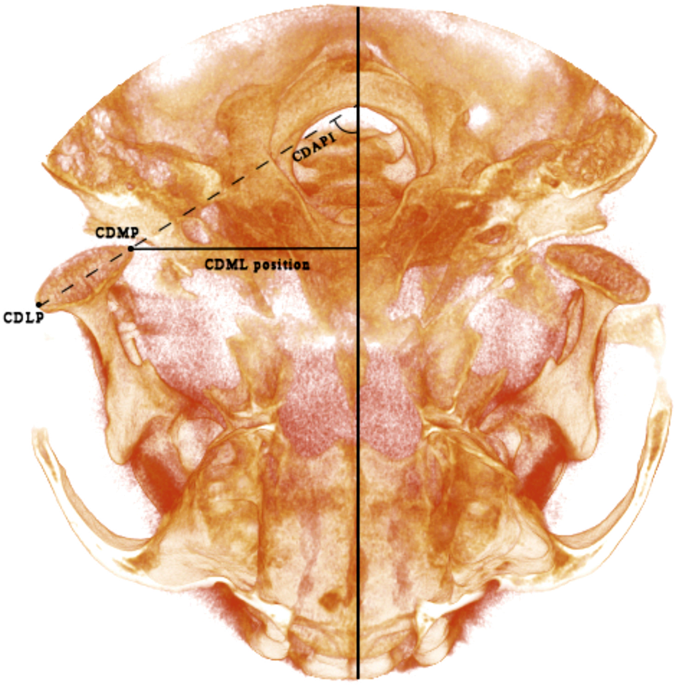

Figure 3

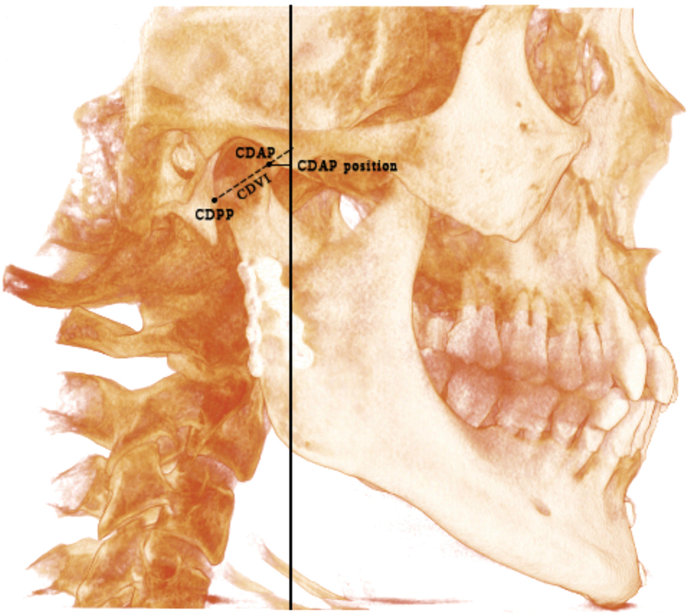

Figure 4

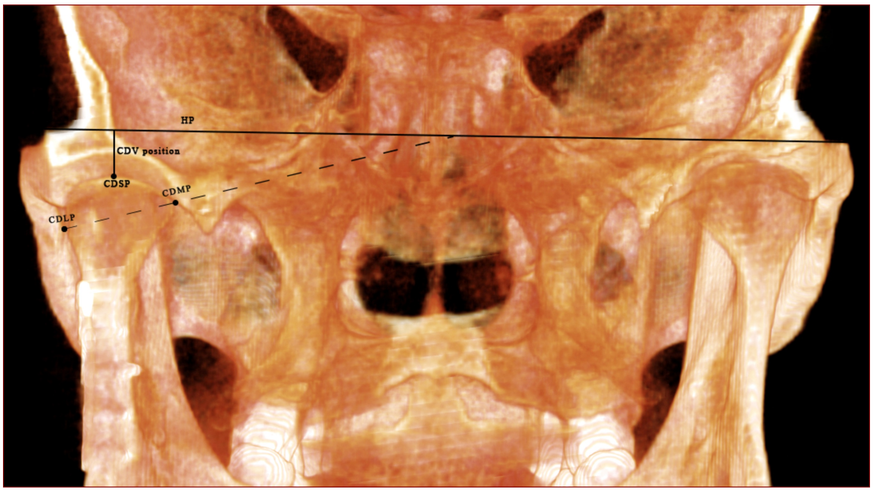

Figure 5

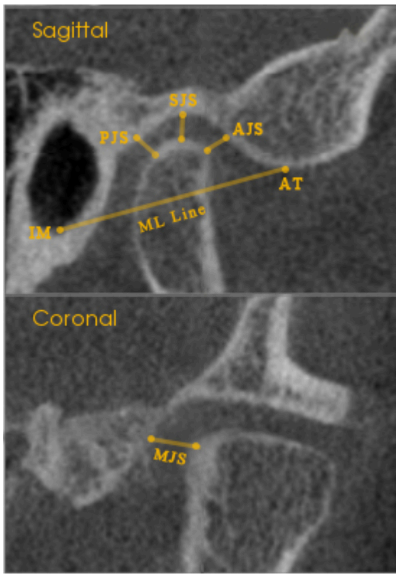

National Institutes of Health Database (PubMed) was conducted for the literature review.

Statistical analysis

Descriptive information was reported as mean and standard deviation. The difference in mean between the two groups was assisted by the independent t-test. Pearson's correlation analyzed the correlation between the joint space, and condylar position and angulation. Also, the relation between the clinical finding and the fracture side measurement was tested. The interclass correlation was utilized to check the agreement between two independent observers. The *P*-value was set at 0.05 or less to indicate the statistical significance. All analysis was performed by the IBM SPSS Statistics version 26 (IBM Corp., Armonk, NY, USA).

Results

Thirty-one patients were included in this study; two of them draped off the follow up, and one of them preferred the conservative treatment upon ORIF. Twenty-nine cohort (16 in the RMA group, 13 in the SMA group) with unilateral subcondylar fracture were consecutively included. Demographics and fractures characteristics are shown in table 1. Of the study population, 23 were male (79.3%), and 6 were female (21.7%). The patient ranged in age from 18 to 69 years, with an average age of 37.2 ± 14.4 years. All patients completed 6 months of follow-up, ranging from 6 months to 16 months. Sixteen patients (55.17%) were treated with RMA, and 13 (44.82%) were treated utilizing the SMA. Both groups were treated consecutively by one surgeon. Intra-operative bleeding was minimal, and none of the patients required a blood transfusion. In four cases of the retromandibular group, the retromandibular vein was sectioned and retracted posteriorly. Operative time in retromandibular patients was shorter than submandibular patients, but the difference was not significant. The parotid fistula was not detected in any case. The facial nerve was encountered in both groups, and temporary weakness was seen in three patients. No permanent facial nerve weakness was detected at the end of the follow-up time. Postoperative malocclusion was found in two patients and was treated by elastic traction. Frey's syndrome, wound infection, abscess, pus discharge, or cellulitis were not detected. Concomitant fractures were treated with a suitable osteosynthesis set.

Helkimo index was conducted to evaluate the patients. In patients with retromandibular approach, AiO was found in 12 patients, whereas the AiI was found in 4 patients. However, the objective clinical finding DiO, I, II were found in 2,10 and 4, respectively. Patients treated with submandibular approach had AiO in 7 patients, while the DiI was found in 5 patients (Table 5).

Table 1. Helkimo index result in fractured patients

Approach	Retromandibular	Submandibular
Ai		
AiO	12	7
AiI	4	5
AiII	0	1
Di		
DiO	2	1
DiI	10	6
DiII	4	5
DiIII	0	1

Radiological assessment

The measurement and parameter between the mean of the fracture and non-fracture side for each approach were tested with an independent *t*-test and listed in table 6, 7.

Table 2, Fracture and non-fracture sides for each approach										
Independent *t*-test										
Acronym	Submandibular approach				P value	Retromandibular approach				P value
	Fracture side		Non-fracture side			Fracture side		Non-fracture side		
	Mean	SD	Mean	SD		Mean	SD	Mean	SD	
CDMLi (HP)	8.4	2	13.3	3	**0.001**	10.7	2	12.2	1.7	**0.03**
CDVi (VP)	57.8	11	60.9	8	0.45	61.7	19	66.6	11.2	0.4
CDAPi (MSP)	64.2	9	76.8	5	**0.001**	72	7	74	6.8	0.42
CDVp	3.3	0.7	2.5	0.6	**0.007**	2.1	0.7	2.7	0.7	**0.01**
CDAPp	7.6	1.7	6.8	3	0.38	7.7	4	7.3	2.5	0.74
CDMLp	46	3.6	49.9	4	**0.02**	52.9	4	50.2	3.3	0.06
AJS	2.5	0.7	2.1	0.7	0.11	2.7	0.6	2.1	0.8	**0.03**
PJS	2.1	0.4	2.4	0.2	0.06	2.2	0.4	2.1	0.4	0.71
MJS	1.8	0.7	2.6	1	**0.04**	2.6	1	2.8	1.2	0.57
SJS	3.2	1.1	2.2	0.7	0.21	2.3	0.7	2	0.6	0.25

Bold is less than 0.05

Table 3. Fracture sides in both approaches was tested by independent t-test					
Approach	Submandibular approach Fracture aide		Retromandibular approach Fracture side		P value
Acronym	Mean	SD	Mean	SD	
CDMi (HP)	8.4	2	10.7	2	**0.02**
CDVi (VP)	57.8	11	61.7	19	0.53
CDAPi (MSP)	64.2	9	72	7	**0.01**
CDVp	3.3	0.7	2.1	0.7	**0.001**
CDAPp	7.6	1	7.7	4	0.98
CDMp	46	3	52.9	4	**0.001**
SJS	3.2	1	2.3	0.7	**0.03**
MJS	1.8	0.7	2.6	1	**0.03**
AJS	2.5	0.7	2.7	0.6	0.58
PJS	1.9	0.6	2.2	0.4	0.19

Bold is less than 0.05

The intercorrelation coefficient between the two independent observers was 8.5, indicating that excellent reliability.

On RMA group, the horizontal, vertical, and midsagittal condylar angulation on the fracture side were (10.7 ± 2 °), (61.7 ± 19 °), and (72 ± 7 °), whereas on the patients with SMA fracture side were (8.4 ± 2 °), (57.8 ± 11 °), and (64.2 ± 9 °), respectively. However, the condylar position to the horizontal plane on RMA was (2.1 ± 0.7 mm) and on SMA patients was (3.3 ± 0.7 mm). The condylar position to the vertical plane in RMA's fracture side was (7.7 ± 4 mm); however, on SMA was (7.6 ± 1.7 mm). In addition, the condylar position to the midsagittal plane on RMA was 52.9 ± 4 mm and on SMA was 46 ± 3.6 mm.

The mediolateral condylar inclination to the horizontal plane on SMA was significantly lower than with RMA (P = 0.02). Furthermore, the anteroposterior condyle inclination to the midsagittal plane was lower on SMA than on the RMA group (P = 0.01). The mediolateral condyle position was higher on the RMA than SMA (P =0.001)

Regarding the joint space, the differences between the fracture sides of the SMA and RMA was tested, the superior, medial, anterior, and posterior joint spaces on SMA patients were 3.2 ± 1 mm, 1.8 ± 0.7 mm, 2.5 ± 0.7 mm, and 1.9 ± 0.6 mm, whereas on RMA's fracture side were 2.3 ± 0.7 mm, 2.6 ± 1 mm, 2.7 ± 0.6 mm and 2.2 ± 0.4 mm.

On the other hand, the joint spaces were tested to find the correlation between condylar position and inclination. The medial joint space was positively related to the condylar mediolateral position in both approaches ($P < 0.05$). However, a positive relation was detected on SMA between the anterior joint space and the anteroposterior condylar position (P = 0.008). Furthermore, the superior joint space had a positive relationship with the vertical condylar position on SMA patients (P = 0.004; Table 8).

Table 4. Joint spaces with different parameters in each approach								
Submandibular approach								
Acronym	AJS		PJS		MJS		SJS	
	Pearson	*P* value	Pearson	*P* value	Pearson	*P* value	Pearson	*P* value
CDMLi (HP)	.142	.643	-.379	.202	-.104	.73	-.211	.489
CDVi (VP)	.546	.054	.216	.479	-.005	.987	.060	.845
CDAPi (MSP)	-.106	.729	-.280	.354	-.231	.447	.067	.828
CDVp	.226	.458	-.068	.824	-.009	.976	.739	**.004**
CDAPp	.699	**.008**	-.085	.782	-.320	.287	.095	.757
CDMLp	.537	.059	.458	.458	.571	**.041**	.142	.644
Retromandibular approach								
CDMLi (HP)	.265	.322	.402	.123	.267	.317	.250	.349
CDVi (VP)	.223	.407	.136	.615	-.596	**.015**	-.535	**.033**
CDAPi (MSP)	-.168	.534	-.040	.884	-.303	.254	.303	.255
CDVp	-.195	.469	-.332	.209	-.303	.255	.275	.303
CDAPp	-.100	.710	-.153	.570	-.111	.683	.291	.274
CDMLp	-.247	.357	-.496	.051	.616	**.011**	.065	.812

Bold is less than 0.05

In comparing the 3D finding and the Helkimo index. A negative relationship was identified between the mediolateral condylar angulation and Helkimo Ai (subjective) and Helkimo Di (objective) on SMA ($P < 0.05$). However, on RMA, the same condylar angulation was significantly related with Helkimo Ai. Furthermore, the mediolateral condylar position had a negative relationship with the Helkimo Ai on the submandibular approach (Table 9).

Table 5. Correlation with clinical outcome								
Approach	Submandibular				Retromandibular			
Acronym	Helkimo Ai		Helkimo Di		Helkimo Ai		Helkimo Di	
	Pearson test	P value	Pearson test	P value	Pearson test	P value	Pearson test	P value
CDMLi (HP)	-.579	**.038**	-.638	**.019**	-.704	**.002**	-.480	.060
CDVi (VP)	-.437	.106	-.364	.221	.219	.414	.400	.125
CDAPi (MSP)	-.293	.332	-.651	**.016**	-.015	.957	-.011	.968
CDVp	-.326	.276	-.410	.164	-.278	.298	.683	**.004**
CDAPp	-.056	.855	.034	.913	.036	.895	-.018	.948
CDMlp	-.611	**.026**	.235	.440	.131	.628	.019	.944
AJS	-.111	.717	.083	.787	-.159	.556	.046	.864
PJS	-.112	.714	-.378	.203	-.331	.210	-.509	**.044**
MJS	.117	.704	.190	.534	-.526	**.036**	-.105	.699
SJS	-.191	.532	-.167	.586	-.290	.277	-.117	.665

Bold is less than 0.05

Discussion

For the subcondylar fracture, the retromandibular approach provides better exposure to the subcondylar fracture region than the other approaches. However, this approach encounters the facial nerve branches; the buccal and marginal mandibular nerve. Despite the meticulous identification of the facial nerve, this method necessitates parotid gland retraction, which may result in facial nerve injury.(15–18)Although the RMA provides ample exposure to the surgical site, the reduction along the buccal surface does not assure a 3-D reduction.(19) On the other hand, the subcondylar fracture line will be approached by the incision below the marginal mandible nerve branch on the submandibular approach. The marginal mandibular nerve is easily retracted within the superior layer of the deep cervical facia. In contrast, other approaches need to identify the facial nerve, which is difficult for inexperienced surgeons. The submandibular approach is straightforward to perform and does not need a virtual learning curve.(10) However, this approach provides less exposure to the submandibular surgical side through the sufficient detachment of the masseter muscle from the posterior border of the mandibular ramus.(10)

Commented [MOU2]: Updated references

Regarding the condylar inclination in both groups, we found the mediolateral condylar inclination to the horizontal plane was significantly lower on the submandibular approach, confirming that the medial inclination of the proximal part, which it is similar to the finding of other studies.(20,21) The anteroposterior condylar inclination to the midsagittal plane was lower than the retromandibular approach. This result is opposite to what Choi et al. found. However, it is in line with previous study.(12) Interestingly, the mediolateral inclination of the condyle to the horizontal plane was significantly correlated, in submandibular approach patients, with both Helkimo Ai and Helkimo Di. This finding indicates the relationship between the mediolateral condyle rotation with the clinical objective and subjective outcome, which is in line with who stated that the rotation of the condyle could be associated with popping sound.

The condyle position to the midsagittal plane was on submandibular patients smaller than on the retromandibular patients $P = 0.02$, which indicates that the fracture stump is located more medially. Additionally,

there was a positive relationship between the medial joint space and medial condylar position. This finding is similar to Hlawitschka et al., who stated that the postsurgical condyle displacement was medial.(22) Briefly, we think that the superior head of the lateral pterygoid muscle and the insufficient exposure of the surgical site can lead to this finding.

In comparing the joint space in submandibular and retromandibular approaches, the medial joint space on submandibular patients was lower than the retromandibular patients, which correlated with mediolateral condylar positional change. However, the other joint space was not significantly different, and there was no relationship between the change in joint space with neither Helkimo Hi nor Helkimo Di.

Although the panoramic radiography is the most popular in dental practice and is frequently used to evaluate the fracture after surgical operation, the CBCTs examination for the condylar fracture provides a three-dimensional view and delineates any fine condylar changes without superimposition nor distortion. Furthermore, the CBCT was found to be more accurate than other methods in determining the condyle points.(23,24) For that, CBCT was used in our study to evaluate the condylar anatomical position.

There are many different evaluation systems to assess the functional impairment of the TMJ, for example, the Mandibular Function Impairment Questionnaire, Craniomandibular Index, and Helkimo Index.(25,26) Helkimo Index has been used to assess the function of the TMJ after the open reduction and internal fixation of the mandibular condylar fracture.(22,27) Furthermore, studies used Helkimo Index to assess the relationship between malocclusion, TMD, and muscle activity.(28,29) Kordass et al. concluded a significant relationship between the popping sound and Helkimo's Di during the mandibular movement.(30) In our study, the Helkimo index was applied as it is reliable and easy to use.(31,32)

Commented [MOU3]: Updated reference

RMA and SMA provide good access to the subcondylar fracture; however, patients' images showed more accurate redaction of the condyle position and angulation in the retromandibular approach. Although the direct access into the surgical field and buccal surface help the surgeon to apply more anatomical reduction and fixation, the complete three-dimensional reduction is still not fully re-established.(33) Moreover, the mediolateral condyle inclination in SMA was related to subjective and objective clinical symptoms, which indicates the rotation of the condyle with the horizontal plane (the path of condylar movement) can be connected to clinical outcome.

The strength of this study was the nature of prospective design and the use of the CBCT to systematically evaluate the accuracy of condylar reduction and compare it with the Helkimo index of the TMJ functional impairment at a six-month follow-up. This can give the surgeon a thought to anticipate the patient outcome related to the CBCT after surgical operation. Furthermore, this study was the first to use three-dimensional CBCT to compare the SMA with RMA concerning the reduction accuracy. This study has limitations. The relatively small sample size in population, and the inhomogeneous distribution of the fracture among the groups might make this study prone to bias. Furthermore, the follow-up time was relatively short, and the CBCT was only taken ten days postoperatively.

Conclusion

The SMA and RMA were systematical radiographically evaluated. The RMA was more able to re-establish the three-dimensional reduction of the subcondylar fracture. In contrast, the SMA was related with decreasing in the mediolateral condylar inclination, which is related to the Helkimo Ai and Helkimo Di outcome.

3. DECLARATIONS

-Funding
The author(s) disclosed receipt of the following financial support for the research, authorship, and/or publication of this article: this study was supported by The National Natural Science Foundation of China (Grant Nos. 81903045 and 82072988); the Guangdong Science and Technology Development Fund (Grant Nos. 2019A1515011867, 2020A1515010405 and 2022A1515012173)。

-Ethical Approval
This study followed the Declaration of Helsinki on medical protocol and ethics and the regional Ethical Review Board of Stomatology College, Lanzhou University approved the study. (No. 201743)

-Consent to Participate
Consent for Publication has been obtained from all participants, and the study was conducted in accordance with the declaration of Helsinki.

-Author Contribution
Conceptualization, Abdo Ahmed Saleh Mohamed, Rao Guangxin, Mai Lianxi, and Karim Ahmed Sakran; Data curation, Mai Lianxi, Karim Ahmed Sakran, Saddam Noman Al-wesabi, and Chaobin Pan; Formal analysis, Abdo Ahmed Saleh Mohamed, and Rao Guangxin; Investigation, Abdo Ahmed Saleh Mohamed and Rao Guangxin; Methodology, Abdo Ahmed Saleh Mohamed, Rao Guangxin, and Karim Ahmed Sakran; Project administration Abdo Ahmed Saleh Ahmed, Rao Guangxin, and Chaobin Pan; Resources, Abdo Ahmed Saleh Mohamed, Rao Guangxin and Mia Lianxi; Software, AbdoAhmed Saleh Mohamed, Karim Ahmed Sakran, and Saddam Noman Al-wesabi; Supervision, Chaobin Pao and Zhaoyu Lin, Validation, Abdo Ahmed Saleh Mohamed, Rao Guangxin; Visualization, Abdo Ahmed Saleh Mohamed, Rao Guangxin and Mai Lianxi; Writing – original draft, Rao Guangxin, Mai Lianxi, and Karim Ahmed Sakran, Saddam Noman Al-wesabi and zhaoyu Lin; Writing – review and editing, Abdo Ahmed Saleh Mohamed, Rao Guangxin, Zhaoyu Lin and Chaobin Pan. All authors have read and agreed to the published version of the manuscript. The author(s) read and approved the final manuscript.

-Competing interests
The authors declare that they have no competing interest

-Availability of data and materials
The datasets used and/or analyzed during this study are available from the corresponding author on reasonable request

Reference

1. Silvennoinen U, Iizuka T, Lindqvist C, Oikarinen K. Different patterns of condylar fractures: an

analysis of 382 patients in a 3-year period. J Oral Maxillofac Surg. 1992/10/01. 1992;50(10):1032–7.
2. Zachariades N, Mezitis M, Mourouzis C, Papadakis D, Spanou A. Fractures of the mandibular condyle: a review of 466 cases. Literature review, reflections on treatment and proposals. J cranio-maxillo-facial Surg Off Publ Eur Assoc Cranio-Maxillo-Facial Surg. 2006 Oct;34(7):421–32.
3. Lindahl L. Condylar fractures of the mandible. I. Classification and relation to age, occlusion, and concomitant injuries of teeth and teeth-supporting structures, and fractures of the mandibular body. Int J Oral Surg. 1977/02/01. 1977;6(1):12–21.
4. Al-Moraissi EA, Ellis III E. Surgical treatment of adult mandibular condylar fractures provides better outcomes than closed treatment: a systematic review and meta-analysis. J Oral Maxillofac Surg. 2015;73(3):482–93.
5. Al-Moraissi EA, Louvrier A, Colletti G, Wolford LM, Biglioli F, Ragaey M, et al. Does the surgical approach for treating mandibular condylar fractures affect the rate of seventh cranial nerve injuries? A systematic review and meta-analysis based on a new classification for surgical approaches. J Craniomaxillofac Surg. 2018/01/18. 2018;46(3):398–412.
6. Kommers SC, Boffano P, Forouzanfar T. Consensus or controversy? The classification and treatment decision-making by 491 maxillofacial surgeons from around the world in three cases of a unilateral mandibular condyle fracture. J Cranio-Maxillofacial Surg. 2015;43(10):1952–60.
7. Singh K, Huang TCT, Meaike JD, Mills AM, Nathan JM, Lettieri SC, et al. The Medial Femoral Condyle Free Flap for Reconstruction of Recalcitrant Defects in the Head and Neck. Ann Plast Surg. 2021;87(3):291–7.
8. Marwan H, Sawatari Y. What Is the Most Stable Fixation Technique for Mandibular Condyle Fracture? J oral Maxillofac Surg Off J Am Assoc Oral Maxillofac Surg. 2019 Dec;77(12):2522.e1-2522.e12.
9. Huang CM, Chan MY, Hsu JT, Su KC. Biomechanical analysis of subcondylar fracture fixation using miniplates at different positions and of different lengths. BMC Oral Health. 2021 Oct;21(1):543.
10. Nam SM, Lee JH, Kim JH. The application of the Risdon approach for mandibular condyle fractures. BMC Surg. 2013;13(1):25.
11. Gupta S, Bansal V, Mowar A, Purohit J, Bindal M. Analysis between Retromandibular and Periangular Transmasseteric Approach for Fixation of Condylar Fracture - A Prospective Study. Ann Maxillofac Surg. 2021/03/13. 2020;10(2):353–60.
12. Mohamed AAS, Abotaleb B, Ahmed Abdulqader A, Hongliang D, Sakran KA, He D. Three-dimensional assessment of accuracy for open reduction and internal fixation of the subcondylar fracture and its implications on the TMJ function. J Craniomaxillofac Surg. 2021/07/05. 2021;
13. Neff A, Cornelius C-P, Rasse M, Torre D, Audigé L. The Comprehensive AOCMF Classification System: Condylar Process Fractures - Level 3 Tutorial. Craniomaxillofac Trauma Reconstr. 2014 Dec 1;7(1_suppl):44–58.
14. El-Beialy AR, Fayed MS, El-Bialy AM, Mostafa YA. Accuracy and reliability of cone-beam computed tomography measurements: Influence of head orientation. Am J Orthod Dentofac Orthop. 2011;140(2):157–65.
15. Nowair IM, Essa EF, Eid MK. A comparative evaluation of retromandibular versus Modified Risdon approach in surgical treatment of condylar fracture. J cranio-maxillo-facial Surg Off Publ Eur Assoc Cranio-Maxillo-Facial Surg. 2022 Mar;50(3):237–45.
16. Manisali M, Amin M, Aghabeigi B, Newman L. Retromandibular approach to the mandibular condyle: a clinical and cadaveric study. Int J Oral Maxillofac Surg. 2003/05/28. 2003;32(3):253–6.
17. Tang W, Gao C, Long J, Lin Y, Wang H, Liu L, et al. Application of modified retromandibular approach indirectly from the anterior edge of the parotid gland in the surgical treatment of condylar fracture. J oral Maxillofac Surg Off J Am Assoc Oral Maxillofac Surg. 2009 Mar;67(3):552–8.
18. Kang DH. Surgical management of a mandible subcondylar fracture. Arch Plast Surg. 2012 Jul;39(4):284–90.
19. Choi B-H, Huh J-Y, Yoo J-H. Computed tomographic findings of the fractured mandibular condyle after open reduction. Int J Oral Maxillofac Surg. 2003;32(5):469–73.
20. Raveh J, Vuillemin T, Lädrach K. Open reduction of the dislocated, fractured condylar process: indications and surgical procedures. J oral Maxillofac Surg. 1989;47(2):120–6.
21. Iizuka T, Lädrach K, Geering AH, Raveh J. Open reduction without fixation of dislocated condylar process fractures: long-term clinical and radiologic analysis. J oral Maxillofac Surg. 1998;56(5):553–61.
22. Hlawitschka M, Loukota R, Eckelt U. Functional and radiological results of open and closed treatment of intracapsular (diacapitular) condylar fractures of the mandible. Int J Oral Maxillofac Surg. 2005;34(6):597–604.
23. Suomalainen A, Vehmas T, Kortesniemi M, Robinson S, Peltola J. Accuracy of linear measurements using dental cone beam and conventional multislice computed tomography. Dentomaxillofacial Radiol. 2008;37(1):10–7.

24. Zain-Alabdeen EH, Alsadhan RI. A comparative study of accuracy of detection of surface osseous changes in the temporomandibular joint using multidetector CT and cone beam CT. Dentomaxillofacial Radiol. 2012;41(3):185–91.
25. Helkimo M. Studies on function and dysfunction of the masticatory system. II. Index for anamnestic and clinical dysfunction and occlusal state. Sven Tandlak Tidskr. 1974/03/01. 1974;67(2):101–21.
26. Fricton JR, Schiffman EL. The craniomandibular index: Validity. J Prosthet Dent. 1987;58(2):222–8.
27. Kolk A, Neff A. Long-term results of ORIF of condylar head fractures of the mandible: A prospective 5-year follow-up study of small-fragment positional-screw osteosynthesis (SFPSO). J Cranio-Maxillofacial Surg. 2015;43(4):452–61.
28. Lauriti L, Motta LJ, de Godoy CHL, Biasotto-Gonzalez DA, Politti F, Mesquita-Ferrari RA, et al. Influence of temporomandibular disorder on temporal and masseter muscles and occlusal contacts in adolescents: an electromyographic study. BMC Musculoskelet Disord. 2014;15(1):123.
29. Munhoz WC, Hsing WT. Interrelations between orthostatic postural deviations and subjects' age, sex, malocclusion, and specific signs and symptoms of functional pathologies of the temporomandibular system: a preliminary correlation and regression study. CRANIO®. 2014;32(3):175–86.
30. Kordass B, Hugger A, Bernhardt O. Correlation between computer-assisted measurements of mandibular opening and closing movements and clinical symptoms of temporomandibular dysfunction. Int J Comput Dent. 2012;15(2):93–107.
31. Su N, Liu Y, Yang X, Luo Z, Shi Z. Correlation between bony changes measured with cone beam computed tomography and clinical dysfunction index in patients with temporomandibular joint osteoarthritis. J Cranio-Maxillofacial Surg. 2014;42(7):1402–7.
32. Leamari VM, Rodrigues A de F, Camino Junior R, Luz JGC. Correlations between the Helkimo indices and the maximal mandibular excursion capacities of patients with temporomandibular joint disorders. J Bodyw Mov Ther. 2019;23(1):148–52.
33. Kolk A, Scheunemann L-M, Grill F, Stimmer H, Wolff K-D, Neff A. Prognostic factors for long-term results after condylar head fractures: A comparative study of non-surgical treatment versus open reduction and osteosynthesis. J Cranio-Maxillofacial Surg. 2020;48(12):1138–45.

4. ACKNOWLEDGEMENTS

The authors thank Dr. Du hongliang, Dr. T. yu, Dr. Che for their support during this study. We would also like to thank our patients and nurses for their patient and support.

Figure legend

Figure 1. Slice locator position in different planes................. **Error! Bookmark not defined.**
Figure 2. Condylar position and inclination to the midsagittal plane in RMA group ..... **Error! Bookmark not defined.**
CDMP; condylar medial point, CDLP; condylar lateral point,
CDAPi (MSP) ; condylar anteroposterior inclination to the midsagittal plane, CDML position; condylar mediolateral position

Figure 3. Condylar position and inclination to the vertical plane in RMA group.......... **Error! Bookmark not defined.**
CDPP; condylar posterior point, CDAP; condylar anterior point, CDVi(VP); condylar vertical inclination to Vertical plane, CDAP position; condylar anterior posterior position

Figure 4. Condylar position and inclination to the horizontal plane in SMA group **Error! Bookmark not defined.**CDLP; condylar lateral point, CDMP; condylar medial point, CDSP; condylar superior position, HP; horizontal plane, CDV position; condylar vertical position, CDMLi (HP); condylar mediolateral inclination to horizontal plane.

Figure 5. Joint spaces.. **Error! Bookmark not defined.**
PJS; posterior joint space, SJS; superior joint space, AJS; anterior joint space, MJS; medial joint space, AT; anterior tubercle, IM; auditory meatus , ML line; tuberculo-meatus line

Tables

Table 1. Demographic data and characteristic of fracture ...... **Error! Bookmark not defined.**
Table 2. Helkimo Ai and Di index score................................ **Error! Bookmark not defined.**
Table 3. Landmarks Definition... **Error! Bookmark not defined.**
Table 4. Planes, lines and measurements' definition.............. **Error! Bookmark not defined.**
Table 5. Helkimo index result in fractured patients .... **Error! Bookmark not defined.**
Table 6. Fracture and non-fracture sides for each approach ... **Error! Bookmark not defined.**
Table 7. Fracture sides in both approaches was tested by independent t test**Error! Bookmark not defined.**
Table 8. Joint spaces with different parameters in each approach ....**Error! Bookmark not defined.**
Table 9. Correlation with clinical outcome .... **Error! Bookmark not defined.**
